# Radiation induced angiosarcoma of the breast: case series and review of the literature

**Published:** 2018-12

**Authors:** Z Amajoud, AS Vertongen, R Weytens, J Hauspy

**Affiliations:** Departement of gynecology and obstetrics, GZA Sint Augustinus, Antwerp;; Co-assistant, UZ Leuven;; Departement of radiotherapy, GZA Sint Augustinus, Antwerp;; Departement of gynecologic oncology, GZA Sint Augustinus, Antwerp

**Keywords:** radiotherapy induced angiosarcoma, breast, case series

## Abstract

Radiation therapy (RT) is an essential adjuvant treatment in early stage breast cancer decreasing the risk of local recurrence. One of the rare late complications of RT is the development of a second primary tumor in the form of radiation-induced angiosarcoma (RIAS). In this report, we present a series of cases of RIAS at a single center and discuss the presentation, management and outcome of this rare iatrogenic malignancy.

We conducted a retrospective data analysis of all diagnosed RIAS at the GZA Sint Augustinus Hospital between 2008 and 2018 (n=10). Additionally, a literature search was done.

The women were between 64 to 86 years old (mean 73 years). Median follow up was 13,0 months [range 6-96 months] The latency period till RIAS ranged from 4.1 to 14.9 years (average 7.3 years). All tumors, with various clinical presentations were located in the radiation field with sizes from 1 to 10 cm. Nine patients had surgery. Disease-free interval for first recurrence of RIAS was 2-51 months (median 4 months). Overall survival for 1, 2 and five years is respectively 80, 69 and 46%. Comparable numbers were found in the literature.

In conclusion, RIAS can occur beyond the conventional 5-year oncological follow-up. Long-term follow-up is necessary with particular attention to post irradiation skin lesions to ensure early detection and prompt therapeutic intervention.

Surgery is the golden standard, however the role of chemotherapy and/or RT remains ambiguous. Further investigation is needed.

## Introduction

Radiation therapy (RT) is an essential adjuvant treatment in early stage breast cancer decreasing the risk of local recurrence, most importantly after breast conserving surgery (Fisher et al., 2002; van Dongen et al., 1992) One of the rare late complications of RT is the development of a second primary tumor in the form of radiation-associated sarcoma (RIAS) close to the RT target volume. In this report, we present a series of cases of radiation-induced angiosarcoma following RT for breast cancer (RIAS-BC) and discuss the presentation, management and outcome of this rare iatrogenic malignancy.

## Patients

We conducted a retrospective data analysis of all diagnosed RIAS treated in GZA Sint Augustinus Hospital between 2008 and 2018. Ten patients with a diagnosis of radiation-induced angiosarcoma following radiation therapy for invasive (9/10) or in situ (1/10) breast cancer were identified.

Patient and treatment characteristics are depicted in Table I. The women, aged between 50-80 years old (mean age: 65 years), presented with early stage breast cancer and were primarily treated surgically. Only one patient underwent a mastectomy with sentinel node biopsy for DCIS. All other patients underwent a lumpectomy with axillary lymph node dissection. One patient underwent a bilateral lumpectomy for bilateral breast cancer.

**Table I:** — Patient characteristics at the moment of diagnosis of breast cancer and treatment.

No	Age at BC Dx (y)	Stage	Histology	Surgery	Adj HT	Adj CT	Adj RT (dose and frequency)
1	64	T1b N0 M0	IDC G1 ER/PR +	R lumpectomy + ALND	Yes	No	R breast 50 Gy x 25 + boost 15 Gy
2	63	T1b N1 M0	IDC G2 ER/PR +	R lumpectomy + ALND	Yes	CMF	R breast 50 Gy x 25 + boost 16 Gy MSP 45 Gy x 25f
3	66	T1c N0 M0	IDC G1 ER/PR +	L lumpectomy + ALND	Yes	No	L breast 50 Gy x 25 + boost 15 Gy
4	68	DCIS	DCIS G3	L mastectomy + sentinel	No	No	L chestwall 50 Gy x 25
5	65	T1c N1 M0	IDC G2 ER/PR -, HER2 +	R lumpectomy + sentinel + ALND	No	CEF, Taxotere, Herceptine	R breast 50 Gy x 25 + boost 16 Gy MSP 45 Gy x 25
6	80	R T1b N0 M0 / L T2 N1 M0	IDC R G2/ L G1 ER/PR +	bilateral lumpectomy + ALND	Yes	No	Bilateral 40 Gy x 15 + boost 16 Gy x 8
7	56	T1c N0 M0	IDC G2 ER/PR +,HER2 +	L lumpectomy + ALND	Yes	CEF, Herceptine	L breast 50 Gy x 15 + boost 16Gy x 8
8	64	T1c N1 M0	IDC G2 ER/PR +	R lumpectomy + ALND	Yes	CEF, Taxotere	R breast IORT 9 Gy + 50 Gy x 25 MSP 45 Gy x 25
9	77	T1b N0 M0	IDC G1 ER/PR +	L lumpectomy + sentinel + ALND	Yes	No	L breast 40 Gy x 15
10	50	T2 N1 M0	IDC G3 ER/PR -	R lumpectomy + ALND	No	CEF	R breast 50 Gy x 25 + boost 10 Gy MSP 45 Gy x 25

(No = case number, BC = breast cancer, Dx = diagnosis, y = years, Adj = adjuvant, HT = hormonal therapy, CT = chemotherapy, RT = radiotherapy, IDC = invasive ductal carconima, DCIS = ductal carcinoma in situ, G = grade, ER = estrogen receptor, PR = progesterone receptor, HER2 = human epidermal growth factor receptor 2, ALND = axillary lymphnode dissection, R = right, L = left, CMF = cyclophosphamide + methotrexate + 5-fluorouracil, CEF = cyclophosphamide + epirubicine + 5-fluorouracil, Gy = gray, MSP = median subclavian and parasternal lymphnode areas, IORT = intra-operative radiotherapy)

All patients received adjuvant RT to the whole breast, total dosage ranging between 40 and 66 Gray (Gy). An additional boost to the tumor bed (15-16Gy) was applied to 8/9 patients treated with breast-conserving surgery. In seven patients the boost was delivered with external beam RT, one patient received an intraoperative boost radiation treatment with a dose of 9 Gy. In one patient no additional boost was done seen age and histological type. Note that the patient that had a mastectomy for DCIS received radiation because of a positive posterior margin.

At time of the diagnosis of RIAS patients were 64-86 years old (mean 73 years). The time between the end of adjuvant radiotherapy and the diagnosis of RIAS ranged from 4.1 to 14.9 years, with an average of 7.3 years.

**Figure 1:**
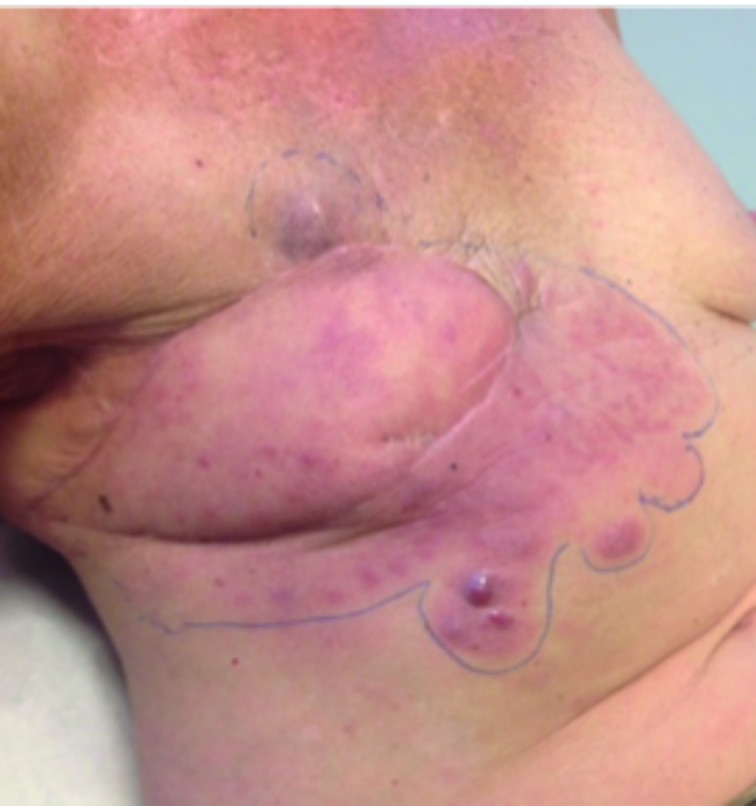
— Presentation of RIAS recurrence status post wide excision with lat dorsi reconstruction and local radiotherapy.

All tumors were located within the radiation fields, with various clinical presentations. Four patients had blue discoloration of the skin, three presented with cutaneous thickening, one had a nodule, another patient developed a rash and finally one patient was diagnosed with a peau d’orange and redness of the skin. Figure 1 shows a recurrence of RIAS with similar features. Tumorsize ranged from 1 to 10 cm.

Initial treatment in nine patients was surgical: four patients underwent a simple mastectomy, a mastectomy with latissimus dorsi flap (LDF) reconstruction was performed on one patient and another one had a mastectomy and thoracic wall resection. The previously mentioned patient with DCIS was treated with a wide local excision followed by LDF reconstruction. One patient received systemic therapy (paclitaxel) due to the presence of RIAS in both breasts. Treatment details for each patient are visualized in Table II.

**Table II:** — Patient characteristics, treatment and surveillance RIAS (radiation induces angiosarcoma).

No	Age at dx (y)	Time from primary ca to AS (y)	Presentation	Initial treatment	Tumor-size (cm)	Recurrence and secondary treatment	Follow up (months)	Latest status
1	72	8.3	Thickening of skin	Simple mastectomy	3.5	1. Local, DFI 51m => Wide excision 2. Local + Distant, DFI 1 m => Re-excision + CT (Paclitaxel => Caelyx)	62	DIED
2	72	9.1	Peau d’orange, redness	Simple mastectomy	7	1. Local, DFI 4 m => RT (20 Gy)	6	DIED
3	70	4.1	Blue discoloration	Simple mastectomy	7	No	96	NED
4	73	4.8	Blue discoloration	Wide excision + latissimus dorsi flap	6	1. Local, DFI 3m => wide excision + skingraft 2. Local, DFI 2m => Left hemi thoracotomy 3. Distant, DFI 2m => CT (Paclitaxel)	13	DIED
5	71	5.7	Thickening of skin	Simple mastectomy	1.5	1. Local, DFI 13m => wide excision with lat dorsi reconstruction 2. Local, DFI 4m => RT (45 Gy) 3. Distant, DFI 5m => CT (Paclitaxel => Caelyx)	32	DIED
6	86	5.6	Blue discoloration	Taxol	10	1. Local, DFI 4m => RT (15Gy)	8	DIED
7	64	8.3	Rash	Wide mastectomy + latissimus dorsi flap	5	No	13	NED
8	71	6.7	Nodule	Simple mastectomy	1	No	13	NED
9	84	6.1	Nodule and small haematomas	Simple mastectomy	2.3	No	12	NED
10	65	14.9	Fibriotic lesion	Wide mastectomy + thoracic wall resection	4.5	No	23	NED

(No = case number, Dx = diagnosis, y = years, AS = angiosarcoma, cm = centimetre, DFI = disease free interval, m = months, RT = radiotherapy, CT = chemotherapy, NED = no evidence of disease).

Median follow up was 13,0 months [range 6-96 months]. Recurrent disease was seen in five out of ten patients treated for RIAS with a median disease-free-interval of 4 months [range 2-51months]. The primary recurrence in all patients was local. Three out of five had a wide local excision of the lesion with or without reconstructive surgery. Two patients had adjuvant radiotherapy (15-20Gy).

A second local recurrence was identified in three cases (3/5) with one patient having distant metastasis at this time. Patient 2 had a re-excision, was started on Paclitaxel and switched thereafter to Caelyx because of progressive disease.

Patient 4 who had a DFI of 2 months after the first recurrence underwent a left hemithoracotomy but relapsed two months later with distant disease and was started on Paclitaxel. Patient 5 received RT after a DFI of 4 months but also recurred after five months and was treated with Paclitaxel and Caelyx.

**Figure 2:**
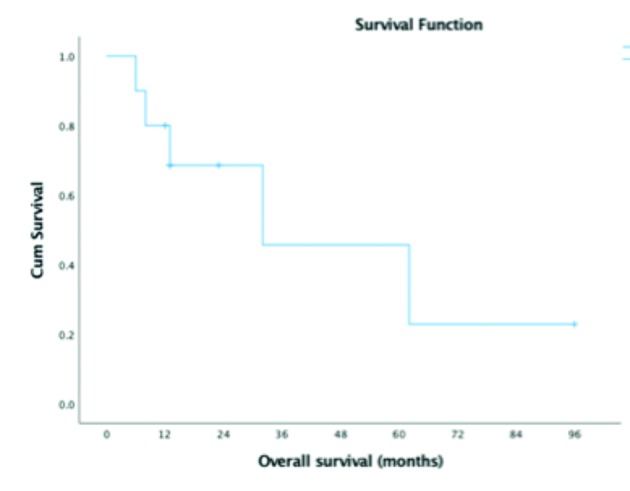
— Kaplan Meier overall survival.

Five patients died of the disease. Five patients continue to be under observation with NED. The overall survival for 1, 2 and five years is respectively 80, 69 and 46%. (Figure 2)

## Discussion

RIS-BC (radiation induced sarcoma after breast cancer) is a rare oncologic entity complicating 0.2% of all irradiated BC’s.(Kirova et al., 2005) In a large series of 13.472 patients, Kirova et al. demonstrated a cumulative incidence of RIS-BC development of 0.07% at 5 years, 0.27% at 10 years and 0.48% at 15 years, confirming the importance of long-term follow-up of BC survivors. To be noted that in this study only 13 out of 35 sarcomas were angiosarcomas. According to Yap et al. (2002), the cumulative incidence of RIAS fifteen years after the initial radiotherapy is 0,09%. In other studies the overall incidence of angiosarcoma varies between 0.002% and 0.050% per year (Cozen et al., 1999; Marchal et al., 1999). However, there are other studies that don’t report the incidence (Torres et al., 2013; Depla et al., 2014; Rombouts et al., 2019). We also don’t report incidence as not all patients treated with radiation for breast cancer in our center had their follow up with us, hence we would under- or overestimate a calculated incidence.

The most significant prognostic factor for recurrence is lesion size at the time of diagnosis, therefore early diagnosis is essential (Jerbi et al., 2007; Depla et al., 2014). Likely due to small sample size, we can’t detect the same statistical significance for tumor size.

Nonetheless, the prognosis remains dismal with a 5-year survival rate varying from 28% to 54% (Vorburger et al., 2005; Torres et al., 2013; Depla et al., 2014; Fraga-Guedes et al., 2015).

The criteria for RIS have been introduced by Cahan et al. (1948) and consist of four determinants for accurate diagnosis: (1) development of the sarcoma in the irradiated field, (2) a latency period of at least 4 years, (3) histologic proof of sarcoma and (4) no evidence of sarcoma in the primary tumor.

For the development of RIS-BC, a latency period has been reported ranging from 3 to 20 years (Cahan et al., 1948; Kirova et al., 2005; Seinen et al., 2012; Torres et al., 2013). Similarly, in this study, we report a time between adjuvant radiotherapy and diagnosis of RIAS ranging from 4.1 to 14.9 years with a mean of 6.4 years.

Our findings are comparable with the literature considering age at diagnosis, latency period and overall survival (Table III).

**Table III:** — Literature review.

Literature review	N	Incidence	Median Age (y)	Tumor size (cm)	Latency (y)	Overall survival % (1-2-5y)
Rombouts	209	0.1%	58 [18-97]	n/a	8 [3-20]	n/a-n/a-40.5
Torres	95	n/a	62 [34-92]	5 [0.2-24]	7 [1.4-26]	91 / 78 / 54
Depla	222	n/a	69 [36-96]	4.5 [0.1-34]	6 [ 1-24]	n/a-n/a-43
GZA	10	n/a	64.5 [50-80]	4.5 [1-10]	6.4 [4.1-14.9]	80 / 69 / 46

(N = number of patients, y = years, cm = centimetre, n/a = non-applicable)

Most patients present with a painless nodule in the radiated field with blueish or purple skin discoloration, sometimes difficult to differentiate from benign, post radiation skin changes or tumor recurrence (Torres et al., 2013; Depla et al., 2014; Uryvaev et al., 2015).

No evident risk factors for RIAS-BC that would require special monitoring in irradiated BC patients have been documented thus far, although higher cumulative doses of irradiation have been suggested to potentiate the development of secondary malignancies (Kirova et al., 2006). Chronic lymphedema appears to be related with a higher risk of sarcoma (Stewart and Treves, 1948) and could be added to the risk assessment in the follow up of breast cancer patients.

Surgical resection with an R0 margin remains the mainstay of treatment for RIAS-BC which may require muscle flap reconstruction or skin graft to cover the soft tissue defect. Most literature refers to R0 as negative margins without further specification. Clear consensus regarding required margins is not reached. A 1 cm margin for small sarcoma tumors is generally accepted, however for angiosarcomas characterized by cutaneous infiltrative pattern a wider margin up to 3 cm is proposed (Pencavel and Hayes, 2009; Al-Benna et al., 2010). It is unclear if chemotherapy or RT have a role, because of the lack of prospective clinical studies, and absence of randomized controlled trials evaluating the efficacy and optimal sequencing of these treatment modalities. A systematic review by Depla et al. (2014) representing 222 patients showed enhanced local control when re-irradiation followed surgery. No advantage on overall survival was seen. Sher et al. (2007) published results on chemotherapy in metastatic setting with a response rate of 48%. However, eighty-one percent of the population (56 of 69 patients) consisted of primary angiosarcoma of the breast which is known to occur in younger patients, with higher risk of concomitant distant metastasis at diagnosis. Radiotherapy-naive tumors may respond differently to chemotherapy than RIAS (Sher et al., 2007). Also Torres et al. (2013) showed lower risk of local recurrence with chemotherapy without benefit in overall survival.

Regarding the use of radiotherapy in our study population, this was only performed in case of a recurrence and not as primary treatment of the RIAS. Chemotherapy was administered in one patient as primary treatment due to inability to operate. Three patients had chemotherapy as treatment of recurrence. No statistically analysis was performed seen small sample size.

In conclusion, the incidence of RIAS is likely to increase due to increased use of RT and longer survival of breast cancer patients. RIAS is a secondary malignancy that can occur beyond the conventional 5-year oncological follow-up. As a result, long-term follow-up is necessary with particular attention to post irradiation skin lesions to ensure early detection and prompt therapeutic intervention.

A clear consensus has not yet been reached regarding the optimal oncological management. Surgery is the golden standard, however the role of chemotherapy and/or RT remains ambiguous. Accordingly, there is an obvious need for a Belgian population-based study landscaping the incidence and characteristics of this rare disease.
